# Siddha formulations for cancer management: A review of preclinical and clinical data

**DOI:** 10.6026/973206300221582

**Published:** 2026-03-31

**Authors:** Abarna Balasubramani, I. Shaira Banu, Jinavani Jeevagan, Suresh Ramasamy, Sathish Adithya Rajathinakaran, Medini Elanchezhian

**Affiliations:** 1 Department of Nanju Maruthuvam (Siddha Toxicology), National Institute of Siddha, (Affiliated with the Tamil Nadu Dr. M.G. R Medical University), Ministry of Ayush, Chennai, Tambaram Sanatorium, Tamil Nadu, India; 2 Department of Gunapadam (Siddha Pharmacology), National Institute of Siddha, (Affiliated with the Tamil Nadu Dr. M.G. R Medical University), Ministry of Ayush, Chennai, Tambaram Sanatorium, Tamil Nadu, India; 3 Department of Siddhar Yoga Maruthuvam, Government Siddha Medical College, (Affiliated with the Tamil Nadu Dr. M.G. R Medical University), Ministry of Ayush, Chennai, Tamil Nadu, India

**Keywords:** Cancer, Siddha medicine, anti-cancer drugs, internal medicine, anti-proliferative drugs

## Abstract

Cancer continues to be a significant public health challenge in India, with 1,461,427 new cases reported in 2022. A review of various Siddha formulations evaluated for anticancer activity reveals promising *in vitro* results, particularly against cervical, breast and liver cancers. MTT assay was used to assess cytotoxicity, with IC_50_ values ranging from 0.075 µg/mL to 1000 µg/mL, indicating considerable variation in potency. Safety assessments, including acute and chronic toxicity studies, were conducted for many of the formulations, although advanced molecular analyses such as Annexin V and gene expression studies were limited. Hence, the therapeutic potential of Siddha medicine in oncology should be realised with further standardisation, detailed mechanistic investigations and well-designed clinical trials.

## Background:

Cancer is a disease characterised by the uncontrolled proliferation of transformed cells that evolve through natural selection [1]. Over the past decade, substantial progress has been made in understanding the disease, particularly in areas such as oncogenesis and the progression to malignant metastasis [2]. The rising prevalence of cancer in developing nations highlights a global shift from infectious diseases toward a higher incidence of non- communicable, chronic illnesses [3]. The disease has become a major global health concern, being one of the leading causes of both morbidity and mortality worldwide [4]. It is responsible for one in six deaths globally, with the World Health Organisation (WHO) predicting that cancer-related deaths will surpass 13 million annually by 2030 [5]. The need for effective treatment strategies to alleviate this burden and improve patient outcomes is urgent. Cancer treatment remains a complex challenge, with traditional approaches such as surgery, chemotherapy and radiotherapy in use, while newer methods such as stem cell therapy, targeted therapy, ablation therapy, nanoparticles, natural antioxidants and ferroptosis-based therapies are emerging [5]. The development of cancer medicines is a key focus in current oncology research. Additionally, there has been a notable increase in the use of Traditional, Complementary and Alternative Medicine (TCAM) among cancer patients, driven by unmet patient needs, rising treatment costs, adverse effects of conventional treatments and the perception that traditional remedies may offer a safer, more affordable contemporary medicine [6, 7]. The Siddha System of Medicine, one of the traditional Indian medical systems alongside Ayurveda and Unani, has long been recognised for its holistic approach. In the Siddha system, the occurrence in the human body in accordance with naadi has been classified into 4448, among them cancer is referred to under various terms such as Tumour (*Kazhalai*), Carbuncle (Pilavai) and Carcinoma (*Vippuruthi*) [8, 9]. Ancient Siddha texts describe Cancer as an undetermined growth (Putru) with specific references to growths that exhibit the symptoms of Carcinoma (*Vippuruthi*) [10]. Therefore, it is of interest to report scientific research made on traditional formulations in Siddha.

## Mechanism of cancer in Siddha:

According to Siddha philosophy, the body's three humors-*Vatham* (Air), *Pitham* (Fire) and *Kabam* (Water) -are believed to govern metabolic and physiological functions. When these humours are out of balance, it leads to disease. In the case of cancer, a reduction in *Pitham*, which regulates metabolism, increases *Kabam* and *Vatham*, leading to uncontrolled tumor growth [11, 12]. In the initial stage, improper dietary habits and lifestyle factors lead to an increase in body heat, resulting in the aggravation of *Pitha kutram*. Excessive intake of Kaba-promoting substances and residence in marshy or damp environments further aggravate the combined *Pitha-Kaba* imbalance, leading to the formation of abnormal swellings or masses. This condition is traditionally referred to as "*Purappadu*". Subsequently, the *Vatha kutram* also becomes vitiated, converting the condition into a tridoshic disorder (*Mukkutra pini*). The impairment of normal Vatha functions disrupts physiological processes and promotes the growth of abnormal, malignant-like tissues resembling cancerous masses. These pathological changes may manifest in both external and internal organs, affecting the seven fundamental tissues (*Sapta Dhatus*) at various sites of the body. Clinically, the condition presents with severe heat, itching, induration, swelling, pain, bleeding, foul-smelling discharge, excessive burning sensation and ulceration. Over time, the disease spreads to adjacent channels and surrounding organs, a process comparable to metastasis in modern medical terminology. Additionally, it leads to multiple systemic complications throughout the body. These systemic manifestations align with the Ayurvedic concepts of *Arbuda* (Neoplastic growth) and *Granthi* (Cyst/Nodular lesion), which describe large, immobile masses and glandular swellings resulting from vitiated Doshas infiltrating muscle, blood and fat tissues [12, 14]. While Ayurvedic texts do not explicitly define cancer as a distinct disease entity, the pathogenesis is understood through the interaction of aberrant Doshas and weakened Dhatus, where conditions such as *Udara* (Ascites), Gulma (abdominal mass) and Vidradhi (abscess) are identified as abdominal tumors with malignant potential [13, 15]. The etiological framework further suggests that environmental toxins and lifestyle factors act as pathogenic agents that initiate these neoplastic processes, a concept paralleled in modern oncology by the identification of genetic drivers such as proto-oncogenes, tumor suppressor genes and DNA repair genes [15, 16]. Contemporary research has validated the efficacy of numerous herbal compounds through systematic screening, thereby supporting the integration of traditional medicinal knowledge with modern scientific methodologies to enhance therapeutic outcomes [15]. Siddha medicine offers a range of herbal, herbo-mineral and metallic formulations that are said to treat various types of Cancer. In clinical oncology settings, complementary therapies such as these are used to improve wellness, enhance the quality of life, alleviate symptoms and mitigate the side effects of conventional treatments. This approach, known as "Integrative Oncology," combines the benefits of conventional cancer therapies with evidence-based complementary treatments. This integration not only promotes safety but also ensures greater accessibility within public healthcare systems [17]. Hence, we highlight the potential of Siddha medicine as a complementary treatment for cancer, focusing on both internal and external anti-cancer medicines as documented in Siddha literature.

## Epidemiology and biological basis of cancer:

Carcinogenesis is a multistage biological process involving initiation, promotion and progression. The initiation stage involves irreversible genetic or epigenetic alterations that provide cells with a growth advantage. Promotion refers to the clonal expansion of initiated cells under the influence of factors such as chronic inflammation and hormonal signaling and remains potentially reversible. Progression involves the accumulation of additional molecular changes, leading to malignant transformation, invasion and metastasis. Understanding these stages is essential for the development of effective cancer prevention and therapeutic strategies [1]. Cancer continues to pose a major public health burden in India, with an estimated 1,461,427 new incident cases reported in 2022, corresponding to a crude incidence rate of 100.4 per 100,000 populations. Epidemiological projections further indicate that approximately one in nine individuals in India is likely to develop cancer during their lifetime [18]. Neoplastic disorders, whether benign or malignant, are systematically classified according to the lineage of cells from which they originate. The vast majority of human malignancies are carcinomas, accounting for nearly 90% of all cancers and arising from epithelial tissues. Sarcomas are comparatively rare solid tumors derived from mesenchymal tissues such as muscle, bone, cartilage and fibrous connective tissue. Haematological malignancies, including leukemias and lymphomas, originate from hematopoietic and lymphoid progenitor cells and contribute to approximately 8% of cancer cases [19].

From a Siddha literature, cancer-like conditions such as *Kazhalai* (Tumor), Pilavai (Carbuncle) and *Vippuruthi* (Carcinoma) are understood as disorders arising from progressive derangement of the three humors (Vali, Azhal and Iyam), leading to abnormal tissue proliferation and systemic imbalance. In line with this traditional understanding, contemporary experimental research has increasingly explored Siddha formulations for their anticancer potential. Among the reported *in vitro* studies (Table 1 to 11 - see PDF), Siddha anticancer formulations have been most extensively investigated in cervical cancer, accounting for 42.1% of the total studies, followed by breast cancer at 23.7%, while oral, lung, prostate, hepatic, ovarian, blood and pancreatic cancers together constitute 34.2% of the reported investigations. A formulation-wise analysis indicates that Thiriphala Chooranam has been examined most frequently across multiple cancer models, reflecting its broad experimental exploration and therapeutic relevance within Siddha oncology. However, due to the inherent biological heterogeneity among different cancer cell lines, direct quantitative comparison of IC_50_ values across models was not undertaken. Instead, a descriptive analytical approach was employed to evaluate the distribution of Siddha formulations across cancer types and to identify the predominant mechanistic pathways reported, such as apoptosis induction, oxidative stress and mitochondrial dysfunction. Similarly, on comparing the anticancer efficacy of Rasa parpam is markedly enhanced when co- administered with Vallarai Nei, compared with its individual administration. Although both formulations possess inherent anticancer activity, their combined use demonstrates superior cytotoxic effects, suggesting an adjuvant-mediated enhancement of therapeutic efficacy. Also, previous studies on *in vivo* animal models and clinical case reports are compiled and represented in Tables 12 and 13 (see PDF). These findings highlight both the growing scientific interest in Siddha anticancer formulations and the need for deeper mechanistic and safety-oriented investigations to strengthen their translational and clinical relevance.

## Pleiotropic effect of thiripala chooranam:

Among the Siddha formulations reviewed, Thiriphala Chooranam emerges as the most consistently investigated formulation across multiple cancer models. It has demonstrated significant cytotoxic and antiproliferative activity against cervical adenocarcinoma (HeLa), endometrial adenocarcinoma (HEC-1-B), breast adenocarcinoma (MCF-7), oral squamous cell carcinoma (KB), lung carcinoma (A549), hepatocellular carcinoma (HepG2), prostate cancer (LNCaP), ovarian cancer (PA-1) and pancreatic cancer cell lines. Beyond simple cytotoxicity, mechanistic studies have revealed its ability to induce apoptosis, regulate cell-cycle arrest, modulate mitochondrial pathways, alter Bax/Bcl-2 ratios, activate caspases and suppress invasive and metastatic behavior. Thiriphala Chooranam is one of the few formulations supported by acute and sub-acute toxicity studies, as well as *in-vitro* tumor models, reinforcing its translational relevance. The polyherbal composition of Thiriphala is rich in tannins, polyphenols, flavonoids and antioxidant compounds, which may underlie its broad-spectrum anticancer activity. Network pharmacology approaches further elucidate that Triphala interacts with multiple cancer-related targets, including PI3K and NF-kB signaling pathways, thereby broadly mediating proliferation and apoptosis [98]. Experimental evidence indicates that triphala exerts marked anti-proliferative and apoptosis-inducing properties against different tumour cell lines and animal models without causing damage to normal cells [6]. This selective cytotoxicity has been attributed to specific phytochemicals such as chebulinic acid, gallica acid, terchebulin etc, which has been identified as a key molecule responsible for maintaining the antitumour efficacy of the formulation through potent anti-proliferative, pro-apoptotic and anti-migratory effects [6]. The molecular mechanism underlying these antineoplastic properties is associated with the modulation of critical signaling cascades, specifically the PI3K/AKT and MAPK/ERK pathways [98]. For instance, chebulinic acid functions as a critical component by inhibiting these pathways to suppress tumor growth and progression [6]. In pancreatic cancer models, Triphala has been shown to inhibit tumor growth through the generation of reactive oxygen species and the subsequent phosphorylation of p53 and ERK, effects that are abrogated by antioxidant pre-treatment [98].

## Mercury based apoptosis:

Among the Rasagenthi-related formulations, the chloroform extract of Rasagenthi Mezhugu demonstrated comparatively greater anticancer efficacy, as indicated by consistently lower IC_50_ values in cervical cancer cell lines when compared with hexane, ethyl acetate and butanol fractions. Across the evaluated cancer models, Rasagenthi Mezhugu exhibited higher cytotoxic sensitivity in cervical cancer cell lines, particularly ME-180, followed by SiHa, whereas breast and prostate cancer cell lines showed relatively moderate responsiveness. Although differential sensitivity across cell lines was observed, direct quantitative comparison among distinct cancer models was not intended. The available *in vitro* evidence suggests that the Rasagenthi-related (Mercury and sulphur based) Siddha formulations have been consistent in their anticancer activity, which is primarily mediated through an apoptosis-mediated mechanism. The consistent observations made in various cancer cell lines suggest that the formulations cause intracellular oxidative stress, resulting in mitochondrial dysfunction and activation of the intrinsic apoptotic pathway. This mechanism is biologically credible and consistent with the established redox properties of mercury because it has greater affinity for thiol group [120]. Increased levels of reactive oxygen species disrupt mitochondrial membrane potential, enhance cytochrome c release, activate caspase pathways and, in some cases, cause DNA damage, further committing cancer cells to programmed cell death.

## Cuproptosis:

Based on the reported data, several Thambiram-based Siddha formulations have been investigated across a wide range of carcinomas. Thambiram, referred to as copper in modern terminology, has gained renewed attention due to recent advances in copper-based nanomedicine. Contemporary studies on copper nanoparticles demonstrate that elevated intracellular copper disrupts cancer cell homeostasis by interacting with lipoylated enzymes of the tricarboxylic acid cycle, leading to protein aggregation, destabilization of iron-sulfur clusters and induction of lethal proteotoxic stress [121]. In addition, copper undergoes redox cycling that promotes excessive reactive oxygen species generation through Fenton-like reactions and glutathione depletion. In tumor cells, which typically exhibit high intracellular glutathione levels, reduction of Cu^2+^ to the more reactive Cu^+^ state further amplifies oxidative stress, overwhelming antioxidant defenses [122]. This sustained redox imbalance results in mitochondrial dysfunction, lipid peroxidation and DNA damage, ultimately inhibiting tumor cell proliferation [123].

## Siddha formulations in anticancer research: scope and trends:

The Siddha anticancer formulations represent a reverse pharmacology approach, where traditional use over time serves as the guide for experimental verification rather than discovery of new formulation. The prominence of herbal, polyherbal and herbo-mineral compounds indicates a systems-based approach to therapy, contrasting with the single-target strategy typical of modern treatments oncology. The present studies together suggest that Siddha formulations have anticancer activity through various biological mechanisms, such as apoptosis, redox disturbances, mitochondrial damage and newly emerging metal-mediated cell death mechanisms. One of the important trends of these studies is the pleiotropic and condition-specific activity of these formulations in various models of cancer, which are consistent with the Siddha concept of cancer as a system disorder rather than a cellular defect. In general, the Siddha anticancer research program exemplifies a reverse pharmacology-based system, where formulations that have been traditionally validated for clinical utility and acceptance serve as the starting point for experimental research. While only a few Siddha formulations have been studied scientifically to date, the Siddha literature reports a much larger pharmacopeia of clinically active anticancer drugs that remain largely unexplored. This situation points to the need for safety evaluation and efficacy testing of these traditional formulations using modern experimental and clinical approaches. Such efforts would not only help to maintain the integrity of the Siddha system but also facilitate the safe integration of into evidence-based oncology.

## Limitations:

Despite promising cytotoxic and mechanistic data, many Siddha formulations lack comprehensive mechanistic validation, standardized toxicity profiling and reproducibility across experimental models. The absence of gene expression analysis, pathway-level interrogation and systematic *in-vitro* confirmation limits translational relevance. Addressing these gaps through integrated molecular, toxicological and bioinformatics approaches are essential for advancing Siddha oncology research responsibly.

## Conclusion:

This review provides a comprehensive overview of the anticancer potential of Siddha formulations as documented in published experimental and clinical studies. Although many formulations have demonstrated promising cytotoxic effects, a substantial proportion of the reported evidence remains limited to preliminary *in-vitro* screening assays, predominantly MTT-based evaluations. While these findings establish initial anticancer activity, they are insufficient on their own to support widespread clinical translation. Systematic mechanistic investigations, gene expression analyses and rigorous safety profiling are essential to elucidate molecular pathways, confirm target specificity and ensure long-term safety. Given that Siddha medicine is fundamentally rooted in the principle of reverse pharmacology, advancing these traditionally used formulations through structured preclinical and clinical research, rather than halting at preliminary screening, is crucial. In the context of increasing global interest in metal-based and nano-enabled cancer therapeutics, systematic exploration of Siddha medicines holds significant potential to contribute to globally acceptable cancer treatment strategies.
